# Supplementary material for: Cryptic diversity in Astyanax (Characiformes: Acestrorhamphidae) from the Magdalena basin, Colombia: Insights from molecular and morphometric evidence
Source: PLoS One. 2026 Sep 18;21(9):e0347004. doi: 10.1371/journal.pone.0347004 (PMC13588355; doi:10.1371/journal.pone.0347004)
Supplement: Supplementary file 2 [file pone.0347004.s001.docx]

S1 Table Microsatellite loci and primer pairs developed and tested for *Astyanax* sp. in this study. Universal Primers. A: GCCTCCCTCGCGCCA, B: GCCTTGCCAGCCCGC, D: CGGAGAGCCGAGAGGTG [38].

| Locus | Motif | Universal Primer + Forward primer | Reverse primer | Universal Primer |
| --- | --- | --- | --- | --- |
| Afa01 | ATT | GCCTTGCCAGCCCGCTCACCCACCTCTATTGTGACC | TCATTAGAGCATAAATTGTACAAAGACC | B |
| Afa02 | ATCT | GCCTTGCCAGCCCGCGAGTTCATTAATGGGCCATCC | TGTGTGGGATGTATACTGTACTGTGC | B |
| Afa03 | ATCT | GCCTTGCCAGCCCGCTTTCATATAATTGTTGAATGCCTGG | TGCCCTATGAAAATAGCTGCC | B |
| Afa04 | ATCT | GCCTTGCCAGCCCGCTCTGTAGCTTGTAGTCTAAGTGGGC | TGCACATTCACCACAGAGTCC | B |
| Afa05 | ATCT | GCCTTGCCAGCCCGCCGGAGGTCAGCTTGAATACG | TTCTTGTATAATTCTTGCATTCTTGG | B |
| Afa06 | ATT | GCCTTGCCAGCCCGCCAAAAGAAGTGGAAATTAATACGGC | TGCTCCCAGGCATAACATAGG | B |
| Afa07 | ATCT | GCCTTGCCAGCCCGCTAATGCATCAAAGCCTGTGC | TTCATATAACATGCATAAGTGTTGC | B |
| Afa08 | ATCT | GCCTTGCCAGCCCGCTGGAAGTGTGGACATTTGTGG | TGTCCTTCTACTTGTGTGGTTGC | B |
| Afa09 | ATCT | GCCTCCCTCGCGCCACCCCAACAAATAGCACTGAAGC | GCTACCTCAGCCTCATAGGTCC | A |
| Afa10 | ATCT | GCCTCCCTCGCGCCATGTCTAGAATTTCCCCTCTGGG | CGCATGTATGAGACTTCCTGC | A |
| Afa11 | TCTG | GCCTCCCTCGCGCCACAACTTCTCCCAGTGGAGCC | AAATCTATCCAAACCAACATGGC | A |
| Afa12 | ATTAG | GCCTCCCTCGCGCCATCTTACAGCGATAACAGTGCTGC | TCAACCACTACCTGGCCTCC | A |
| Afa13 | ATT | GCCTCCCTCGCGCCATTGCCATAGACATAATCCTTCCC | AAGAGCAGCTCCAGGAGACC | A |
| Afa14 | ATT | GCCTCCCTCGCGCCAACTGCCGAAACACTCTCTGG | TCATCTGTAATACGTCCATCACC | A |
| Afa15 | ATCT | GCCTCCCTCGCGCCATACAGCAATGCTCCAAAACG | CCATTAAATTGGATGAATTTGC | A |
| Afa16 | ATCT | GCCTCCCTCGCGCCAAATAATGGCGCAAATCAATGG | CACTCTCTCTGGCTGTGTTCC | A |
| Afa17 | ATCT | CGGAGAGCCGAGAGGTGAGACTCTGAGTTGAACCCTGC | TGTTCCTAAATTTCCTTCGGG | D |
| Afa18 | ATTAC | CGGAGAGCCGAGAGGTGGAAGGGCTGTGTTCCTGTCC | AGACCATGTACCACCAATGACC | D |
| Afa19 | TCCTG | CGGAGAGCCGAGAGGTGGGCCTGGATCTGAAGTCTCC | TGCAGTGACAAAAGTACAGGGG | D |
| Afa20 | AGGCT | CGGAGAGCCGAGAGGTGGCCCAAAATCAGTCCAGTTAGC | CTACAGAGAAACGGCGGAGC | D |
| Afa21 | ATCT | CGGAGAGCCGAGAGGTGGCACCCAGAAGGTAGTGTTGG | ACCCCACACCATGACAAACC | D |
| Afa22 | ATCT | CGGAGAGCCGAGAGGTGTGCACACATGCCTAAATTTCC | CACACACACATTGAGGCAGC | D |
| Afa23 | ATCT | CGGAGAGCCGAGAGGTGTCTGCTCCCAGTACCACACC | GAAGACTGGAACCACAATCGC | D |
| Afa24 | ATCT | CGGAGAGCCGAGAGGTGATGTTGTTGCTGCTGGATGC | TCACGTCCTACTCCATCAACG | D |
| Afa25 | ATCT | CAGGACCAGGCTACCGTGGAGTGCCCGTCTTATAGTGCC | ACATCATGCACAGCAACTGG | C |
| Afa26 | ATT | CAGGACCAGGCTACCGTGGATACACCTCCATGCCTCAGC | CCCCTTGTGTTAGGATTGGC | C |
| Afa27 | ATT | CAGGACCAGGCTACCGTGCCCACATGAGAGAAAACCACC | TCTGTTGTGGTGAGTGTGCG | C |
| Afa28 | ATCT | CAGGACCAGGCTACCGTGTCCAGCACTGTGAACCACG | GCTGGATGTCCAAACTTCACG | C |
| Afa29 | ATCT | CAGGACCAGGCTACCGTGCCACCTGTTGGCTGAAAACC | CATGCAATGTGACCAGGACC | C |
| Afa30 | ATT | CAGGACCAGGCTACCGTGATACCCAGGACCCCACAGG | GAGAATCACGACCCACCACC | C |
| Afa31 | ATCAG | GCCTTGCCAGCCCGCGACACCACAGCAAACCATGC | AAGATTGGACAGCTGCCTCC | B |
| Afa32 | AAGCT | GCCTTGCCAGCCCGCCACTGTACATCACTGGGCAGC | GTTGCGATCTATTGTCCATGC | B |
| Afa33 | ATATT | GCCTTGCCAGCCCGCACACTGGCGCAGTGGTAGC | TCGCTTATACAGACAGCTCCG | B |
| Afa34 | TGC | GCCTTGCCAGCCCGCTGGGAAATGAAGCTGAAAACG | CACTGAACAGTGCCACTCGC | B |
| Afa35 | ATC | GCCTTGCCAGCCCGCTCAGGATGAGGAGACCCAGG | TGTCCTTCAGCTTCACCTCG | B |
| Afa36 | TCC | GCCTTGCCAGCCCGCCCGATCTGTCTATCCGGAGG | CTGATTCTCCTCCACGGTGC | B |
| Afa37 | ATC | GCCTTGCCAGCCCGCCAGTCGTGGCCTAACAGTCG | GAGATCATCATGTTGTGCCG | B |
| Afa38 | ATC | GCCTCCCTCGCGCCAGCATGAATCATTGCACCTAGC | GGTGCCTCTGTGCATGTAGC | A |
| Afa39 | ATC | GCCTCCCTCGCGCCAGGTCTGCTTGTTCCTCCAGC | TCACACTGGTCAGCCATTCC | A |
| Afa40 | TCC | GCCTCCCTCGCGCCATGGTACACACAGCGTAATGGG | GAATCATGCTAGCTCTGTAAATTGC | A |
| Afa41 | TGC | GCCTCCCTCGCGCCATTGCACTCTCAATACATCGGC | AAGCGTCCCTCTACACAGGC | A |
| Afa42 | TGC | GCCTCCCTCGCGCCAAAGGGAGCATTCAGAGAAGG | CATATCGTCATATTGCCCAGC | A |
