# Supplementary material for: Cryptic diversity in Astyanax (Characiformes: Acestrorhamphidae) from the Magdalena basin, Colombia: Insights from molecular and morphometric evidence
Source: PLoS One. 2026 Sep 18;21(9):e0347004. doi: 10.1371/journal.pone.0347004 (PMC13588355; doi:10.1371/journal.pone.0347004)
Supplement: Supplementary file 3 [file pone.0347004.s006.docx]

Response to the Editor and Reviewers

Manuscript ID: PONE-D-26-14913

Title: Cryptic diversity in *Astyanax* (Characiformes: Acestrorhamphidae) from the Magdalena basin, Colombia: Insights from molecular and morphometric evidence

Dear Dr. Nogueira,

We sincerely appreciate the careful evaluation of our manuscript and the detailed and constructive comments provided by the Academic Editor and the reviewers. We have revised the manuscript accordingly, and we believe that these changes have substantially improved its clarity and scientific quality.

Below, we provide a point-by-point response to all comments. The reviewers’ and editor’s comments are reproduced in bold, followed by our responses. All changes are indicated in the revised manuscript with track changes.

We hope that the revised manuscript is now suitable for publication in PLOS ONE.

Sincerely,

Edna

---

**Responses to the Journal Requirements**

Journal Requirement 1: Please ensure that your manuscript meets PLOS ONE’s style requirements, including those for file naming.

Response: We have reviewed the manuscript and accompanying files to ensure compliance with PLOS ONE’s formatting and file-naming requirements.

Journal Requirement 2: Please review PLOS ONE’s guidelines on sharing author-generated code and ensure that the code supporting the findings is made available without restrictions.

Response: No original author-generated code was developed specifically for this study.

Journal Requirement 3: Please provide an amended Funding Statement declaring all funding or sources of support and include the statement: “There was no additional external funding received for this study.”

Response: We have amended the Funding Statement and included the complete updated wording in the cover letter, as requested. The revised Funding Statement reads:

“This study was supported by a grant framed under the project ‘Variabilidad genética de un banco de peces de los sectores medio y bajo del río Cauca’ (CT-2019-000661), funded by Empresas Públicas de Medellín and Universidad Nacional de Colombia, Sede Medellín. The funders had no role in the study design, data collection and analysis, decision to publish, or preparation of the manuscript. There was no additional external funding received for this study.”

Journal Requirement 4: Figure 1 appears to contain map or satellite imagery that may be copyrighted. Please provide permission or replace the figure with one that complies with the CC BY 4.0 license.

Response: We have replaced Figure 1 with a new map prepared in QGIS using nonproprietary and appropriately licensed or public-domain geographic data. The revised figure does not contain Google Maps, Google Earth, proprietary satellite imagery, or proprietary basemaps.

We also revised the figure legend to identify the geographic data sources as follows:

“Fig 1. Geographic location of the sampling sectors in the Cauca River basin, Colombia. Blue lines represent upstream and dam-proximate sectors (S1, PHI, S2, S3, and S4), in which genetic stock Asp1 predominated, while orange lines indicate downstream sectors (S5, S6, S7, and S8), in which genetic stock Asp2 predominated (see Results). Map layers: Colombia boundary from DANE Marco Geoestadístico Nacional (2024), available at https://geoportal.dane.gov.co/servicios/descarga-y-metadatos/datos-geoestadisticos/; inset boundaries from Natural Earth Admin 0 Countries (scale 1:10m), available at https://www.naturalearthdata.com/downloads/10m-cultural-vectors/10m-admin-0-countries/; rivers from HydroRIVERS v1.0/HydroSHEDS (15 arc-second resolution), available at https://www.hydrosheds.org/products/hydrorivers; elevation and hillshade from the HydroSHEDS void-filled DEM for South America (3 arc-second resolution), available at https://www.hydrosheds.org/hydrosheds-core-downloads; and physical background from Natural Earth II high-resolution raster data (scale 1:10m), available at https://www.naturalearthdata.com/downloads/10m-raster-data/10m-natural-earth-2/. Map created in QGIS without proprietary satellite imagery or basemaps.”

Journal Requirement 5: Please evaluate any references recommended by the reviewers and cite them only if they are relevant.

Response: We evaluated all publications recommended by the reviewers and incorporated those that were directly relevant to the interpretation and discussion of our results. The corresponding changes are described in our responses to Reviewer 1 below.

Journal Requirement 6: Please review the reference list to ensure that it is complete and correct and address any retracted publications.

Response: We carefully reviewed the reference list for completeness and accuracy. We also verified the status of the cited publications and found no retracted articles requiring removal or specific justification.

---

Response to the Academic Editor

The manuscript was evaluated by two expert reviewers, both of whom recognized the relevance of the research question, the robustness of the methodological approaches employed, and the overall quality of the results presented. Before a final decision can be made, the authors should address the comments and suggestions provided by the reviewers.

Response: We thank the Academic Editor for the positive assessment of our study. We have carefully considered and addressed all comments raised by the reviewers. The manuscript has been revised accordingly, and a detailed description of each modification is provided below.

---

Responses to Reviewer 1

We sincerely thank Reviewer 1 for the careful evaluation of our manuscript, the positive assessment of its scientific contribution, and the constructive suggestions.

Comment 1: In relation to the mitochondrial COI similarity between A. microlepis and Asp1, I suggest that the authors include this possibility in the Discussion. This would bring more robustness to the results. Barcoding analyses do not always provide conclusive results in morphologically cryptic species and complex groups such as Astyanax and Psalidodon. Low COI divergence may result from recent evolutionary radiation and other taxonomic and evolutionary processes.

Response: We agree with the reviewer that the mitochondrial similarity between A. microlepis and Asp1 deserves further consideration. We have expanded the Discussion to acknowledge that low COI divergence among closely related lineages may result from relatively recent evolutionary radiation and incomplete sorting of mitochondrial lineages.

The revised text reads:

“Consistent with this interpretation, low mitochondrial divergence based on COI barcodes has been reported among closely related lineages of Astyanax, a pattern that may reflect relatively recent evolutionary radiation and incomplete sorting of mitochondrial lineages [17].”

We evaluated the references suggested by the reviewer and incorporated those that were directly relevant to Astyanax and to the interpretation of our results.

Comment 2 — Line 61: "this is not the correct reference"

Response: The text has been corrected as suggested. The revised reference 8 in the list is:

“Toledo-Piza M, Baena EG, Dagosta FCP, Menezes NA, Ândrade M, Benine RC, et al. Checklist of the species of the Order Characiformes (Teleostei: Ostariophysi). Neotrop Ichthyol. 2024;22(1). doi:10.1590/1982-0224-2023-0086.”

Comment 3 — Line 71: "s in Astyanax´s, not in italic"

Response: The text has been corrected as suggested.

Comment 4: The name *Psalidodon fasciatus* should be used only for populations from the type locality in the São Francisco River basin.

Response: We thank the reviewer for this important clarification. We agree that the name *Psalidodon fasciatus* should be restricted to populations from its type locality in the São Francisco River basin.

Our original sentence was intended to describe the geographic distribution of species currently assigned to *Psalidodon* as a whole, rather than to suggest that *P. fasciatus* occurs throughout all the river basins mentioned. To remove this ambiguity, we revised the passage as follows:

“This species had traditionally been identified as *Astyanax fasciatus* [7] until this nominal species was reallocated to the recently resurrected genus *Psalidodon*. Species currently assigned to *Psalidodon* are geographically restricted to South American river basins, including the Rio São Francisco, the type locality of *P. fasciatus*, and the Paraná–Paraguay basin [2,5].”

---

Responses to Reviewer 2

We sincerely thank Reviewer 2 for the positive assessment of our manuscript and for the helpful suggestions.

Comment 1: Page 11, line 50: The genus does not occur as far south as Patagonia. The southernmost records of Astyanax, under its current circumscription, are A. abramis and A. lacustris in the Río de la Plata estuary in Argentina.

Response: We thank the reviewer for identifying this geographic inaccuracy. We have revised the sentence accordingly. It now reads:

“...extending from Texas to the Río de la Plata estuary in Argentina.”

Comment 2: Page 28, second paragraph, line 8: Change “configuration” to “shape.”

Response: We have replaced “configuration” with “shape,” as suggested.

Comment 3: Page 44, Figure 2: The quality of the figure is not good and needs to be improved.

Response: We have replaced Figure 2 with a higher-resolution version and improved the legibility of its labels and graphical elements.
