# Supplementary material for: Cryptic diversity in Astyanax (Characiformes: Acestrorhamphidae) from the Magdalena basin, Colombia: Insights from molecular and morphometric evidence
Source: PLoS One. 2026 Sep 18;21(9):e0347004. doi: 10.1371/journal.pone.0347004 (PMC13588355; doi:10.1371/journal.pone.0347004)
Supplement: Peer review history

# Peer review: Decision Letter 0

**DOI:** 10.1371/journal.pone.0347004.r001

**Authors:** Caio Nogueira

22 Jun 2026

Dear Dr. Márquez,

Thank you for submitting your manuscript to PLOS ONE. After careful consideration, we feel that it has merit but does not fully meet PLOS ONE’s publication criteria as it currently stands. Therefore, we invite you to submit a revised version of the manuscript that addresses the points raised during the review process.

Please submit your revised manuscript by Aug 06 2026 11:59PM. If you will need more time than this to complete your revisions, please reply to this message or contact the journal office at plosone@plos.org. When you're ready to submit your revision, log on to https://www.editorialmanager.com/pone/ and select the 'Submissions Needing Revision' folder to locate your manuscript file.

- A letter that responds to each point raised by the academic editor and reviewer(s). You should upload this letter as a separate file labeled 'Response to Reviewers'.
- A marked-up copy of your manuscript that highlights changes made to the original version. You should upload this as a separate file labeled 'Revised Manuscript with Track Changes'.
- An unmarked version of your revised paper without tracked changes. You should upload this as a separate file labeled 'Manuscript'.

If you would like to make changes to your financial disclosure, please include your updated statement in your cover letter. Guidelines for resubmitting your figure files are available below the reviewer comments at the end of this letter.

If applicable, we recommend that you deposit your laboratory protocols in protocols.io to enhance the reproducibility of your results. Protocols.io assigns your protocol its own identifier (DOI) so that it can be cited independently in the future. For instructions see: https://journals.plos.org/plosone/s/submission-guidelines#loc-laboratory-protocols. Additionally, PLOS ONE offers an option for publishing peer-reviewed Lab Protocol articles, which describe protocols hosted on protocols.io. Read more information on sharing protocols at https://plos.org/protocols?utm_medium=editorial-email&utm_source=authorletters&utm_campaign=protocols.

As the corresponding author, your ORCID iD is verified in the submission system and will appear in the published article. PLOS supports the use of ORCID, and we encourage all coauthors to register for an ORCID iD and use it as well. Please encourage your coauthors to verify their ORCID iD within the submission system before final acceptance, as unverified ORCID iDs will not appear in the published article. *Only*  the individual author can complete the verification step; PLOS staff *cannot*  verify ORCID iDs on behalf of authors.

We look forward to receiving your revised manuscript.

Kind regards,

Caio Santos Nogueira, PhD

Academic Editor

PLOS One

Journal Requirements:

When submitting your revision, we need you to address these additional requirements.

1. Please ensure that your manuscript meets PLOS ONE's style requirements, including those for file naming. The PLOS ONE style templates can be found at

https://journals.plos.org/plosone/s/file?id=wjVg/PLOSOne_formatting_sample_main_body.pdf and

https://journals.plos.org/plosone/s/file?id=ba62/PLOSOne_formatting_sample_title_authors_affiliations.pdf.

2. Please note that PLOS One has specific guidelines on code sharing for submissions in which author-generated code underpins the findings in the manuscript. In these cases, we expect all author-generated code to be made available without restrictions upon publication of the work. Please review our guidelines at https://journals.plos.org/plosone/s/materials-and-software-sharing#loc-sharing-code and ensure that your code is shared in a way that follows best practice and facilitates reproducibility and reuse.

3. Thank you for stating in your Funding Statement:

“This study was supported by a grant framed under the Project “Variabilidad genética de un banco de peces de los sectores medio y bajo del río Cauca” (CT-2019-000661, Empresas Públicas de Medellín and Universidad Nacional de Colombia, Sede Medellín). Funders do not play any role in the study design, data collection and analysis, decision to publish, or preparation of the manuscript “\

Please provide an amended statement that declares *all* the funding or sources of support (whether external or internal to your organization) received during this study, as detailed online in our guide for authors at http://journals.plos.org/plosone/s/submit-now.  Please also include the statement “There was no additional external funding received for this study.” in your updated Funding Statement.

Please include your amended Funding Statement within your cover letter. We will change the online submission form on your behalf.

4. We note that Figure 1 in your submission contain [map/satellite] images which may be copyrighted. All PLOS content is published under the Creative Commons Attribution License (CC BY 4.0), which means that the manuscript, images, and Supporting Information files will be freely available online, and any third party is permitted to access, download, copy, distribute, and use these materials in any way, even commercially, with proper attribution. For these reasons, we cannot publish previously copyrighted maps or satellite images created using proprietary data, such as Google software (Google Maps, Street View, and Earth). For more information, see our copyright guidelines: http://journals.plos.org/plosone/s/licenses-and-copyright.

We require you to either (1) present written permission from the copyright holder to publish these figures specifically under the CC BY 4.0 license, or (2) remove the figures from your submission:

a. You may seek permission from the original copyright holder of Figure 1 to publish the content specifically under the CC BY 4.0 license.

We recommend that you contact the original copyright holder with the Content Permission Form (http://journals.plos.org/plosone/s/file?id=7c09/content-permission-form.pdf) and the following text:

“I request permission for the open-access journal PLOS ONE to publish XXX under the Creative Commons Attribution License (CCAL) CC BY 4.0 (http://creativecommons.org/licenses/by/4.0/). Please be aware that this license allows unrestricted use and distribution, even commercially, by third parties. Please reply and provide explicit written permission to publish XXX under a CC BY license and complete the attached form.”

Please upload the completed Content Permission Form or other proof of granted permissions as an "Other" file with your submission.

In the figure caption of the copyrighted figure, please include the following text: “Reprinted from [ref] under a CC BY license, with permission from [name of publisher], original copyright [original copyright year].”

b. If you are unable to obtain permission from the original copyright holder to publish these figures under the CC BY 4.0 license or if the copyright holder’s requirements are incompatible with the CC BY 4.0 license, please either i) remove the figure or ii) supply a replacement figure that complies with the CC BY 4.0 license. Please check copyright information on all replacement figures and update the figure caption with source information. If applicable, please specify in the figure caption text when a figure is similar but not identical to the original image and is therefore for illustrative purposes only.

The following resources for replacing copyrighted map figures may be helpful:

USGS National Map Viewer (public domain): http://viewer.nationalmap.gov/viewer/

The Gateway to Astronaut Photography of Earth (public domain): http://eol.jsc.nasa.gov/sseop/clickmap/

Maps at the CIA (public domain): https://www.cia.gov/library/publications/the-world-factbook/index.html and https://www.cia.gov/library/publications/cia-maps-publications/index.html

NASA Earth Observatory (public domain): http://earthobservatory.nasa.gov/

Landsat: http://landsat.visibleearth.nasa.gov/

USGS EROS (Earth Resources Observatory and Science (EROS) Center) (public domain): http://eros.usgs.gov/#

Natural Earth (public domain): http://www.naturalearthdata.com/

5. If the reviewer comments include a recommendation to cite specific previously published works, please review and evaluate these publications to determine whether they are relevant and should be cited. There is no requirement to cite these works unless the editor has indicated otherwise.

6. Please review your reference list to ensure that it is complete and correct. If you have cited papers that have been retracted, please include the rationale for doing so in the manuscript text, or remove these references and replace them with relevant current references. Any changes to the reference list should be mentioned in the rebuttal letter that accompanies your revised manuscript. If you need to cite a retracted article, indicate the article’s retracted status in the References list and also include a citation and full reference for the retraction notice.

Additional Editor Comments (if provided):

Dear Authors,

The manuscript was evaluated by two expert reviewers, both of whom recognized the relevance of the research question, the robustness of the methodological approaches employed, and the overall quality of the results presented. Both reviewers considered the study to be an important contribution to the understanding of cryptic diversity and diversification processes within Astyanax, one of the most taxonomically challenging groups among Neotropical fishes.

After carefully considering the reviewers’ comments and conducting my own evaluation of the manuscript, I believe that the study has sufficient scientific merit for publication. However, before a final decision can be made, I ask that the authors address the few comments and suggestions provided by the reviewers.

Therefore, the editorial decision for this manuscript is Minor Revision. I kindly request that the authors submit a revised version of the manuscript together with a detailed response letter explaining how each reviewer comment has been addressed.

I congratulate the authors on their work and look forward to receiving the revised version of the manuscript.

[Note: HTML markup is below. Please do not edit.]

Reviewers' comments:

Reviewer's Responses to Questions

**Comments to the Author**

1. Is the manuscript technically sound, and do the data support the conclusions?

Reviewer #1: Yes

Reviewer #2: Yes

**********

2. Has the statistical analysis been performed appropriately and rigorously?

Reviewer #1: Yes

Reviewer #2: Yes

**********

3. Have the authors made all data underlying the findings in their manuscript fully available?

The PLOS Data policy

Reviewer #1: Yes

Reviewer #2: Yes

**********

4. Is the manuscript presented in an intelligible fashion and written in standard English?

Reviewer #1: Yes

Reviewer #2: Yes

**********

Reviewer #1: The manuscript “Cryptic diversity in Astyanax (Characiformes: Acestrorhamphidae) from the Magdalena basin, Colombia: Insights from molecular and morphometric evidence” from Márquez et al. designed and evaluated a set of microsatellite loci to assess genetic diversity and population structure of Astyanax sp. in the middle and lower sections of the Cauca River. Additionally, COI and rag2 sequences were compared within a broad phylogenetic framework, to determine whether groups potentially identified through microsatellites correspond to divergent lineages within the genus, and additionally geometric morphometric analyses were conducted to investigate potential drivers of diversification in Astyanax. The MS by Márquez et al. presents the conditions and quality of literature review and adjusted the scope of this journal. Science is good and the MS is competently presented. The theoretical grounding as well as the proposed methodology are relevant and appropriate. I have made a few corrections throughout the text errors (pointed out in the attached document) that do not depreciate anywhere the results and discussion. In my opinion, the paper is worthily and should be accepted after adjustments.

In relation to the mitochondrial (COI) similarity between A. microlepis and Asp1, I suggest to the authors include in discussion this point of view/possibility. This would bring more robustness to their results.

Barcoding analyses for species identification have been widely used with effective results in several fish species; however, it does not always bring concise results, especially in morphologically cryptic species and complex groups like Astyanax and Psalidodon. Taxonomic uncertainties may result from a combination of aspects such as rapid speciation, species with a wide geographic distribution, lack of descriptions of local morphotypes, and inadequate phylogenetic analyses. Low genetic diversity using DNA barcoding (COI sequences) in Astyanax and Psalidodon (formerly referred to as Astyanax) may be the result of the recent evolutionary process of radiation. (see Ornelas-García et al. 2008 - https://doi.org/10.1186/1471-2148-8-340; Pereira et al. 2011 - https://doi.org/10.3109/19401736.2011.588213; Gavazzoni et al. 2023 - https://doi.org/10.1093/biolinnean/blac139; Gavazzoni et al. 2024 - https://doi.org/10.1089/zeb.2024.0; Silva et al 2024 - https://doi.org/10.1590/1982-0224-2024-0069)

Reviewer #2: That's an very interesting contribution, dealing with the genus Astyanax in the río Magdalena basin in Colombia. The genus Astyanax is one of the most taxonomically-challenging genera among Neotropical freshwater fishes, with a high diversity but relatively little morphological variation across Central and South America. The present MS showcases a particularly complex example, showing that phenotypic divergence in the genus may not be coupled with genetic divergence. The MS is overall very good and I have just very few suggestions to make, which follows below:

1) P. 11, l. 50: The genus does not occurs as far south as Patagonia. The southernmost record for the genus, in its present circunscripion, is the occurrence of Astyanax abramis and A. lacustris in the estuary of the Río de La Plata in Argentina. Some species formerly assigned to the genus occurs in the Pampasic area to the south, but these species were transferred to the genus Psalidodon (e.g., Psalidodon pampa).

2) P. 28, second paragraph (line 8): Change "configuration" for "shape"

3) P. 44, fig. 2: the quality of the figure is not good, it needs to be improved.

**********

what does this mean?). If published, this will include your full peer review and any attached files.

If you choose “no”, your identity will remain anonymous but your review may still be made public.

**Do you want your identity to be public for this peer review?**  For information about this choice, including consent withdrawal, please see our Privacy Policy

Reviewer #1: No

Reviewer #2: No

**********

[NOTE: If reviewer comments were submitted as an attachment file, they will be attached to this email and accessible via the submission site. Please log into your account, locate the manuscript record, and check for the action link "View Attachments". If this link does not appear, there are no attachment files.]

To ensure your figures meet our technical requirements, please review our figure guidelines: https://journals.plos.org/plosone/s/figures

You may also use PLOS’s free figure tool, NAAS, to help you prepare publication quality figures: https://journals.plos.org/plosone/s/figures#loc-tools-for-figure-preparation.

NAAS will assess whether your figures meet our technical requirements by comparing each figure against our figure specifications.

Attachment Submitted filename: PONE-D-26-14913_reviewer.pdf

# Author comment: Author response to Decision Letter 1

**DOI:** 10.1371/journal.pone.0347004.r002

4 Aug 2026

Response to the Editor and Reviewers

Manuscript ID: PONE-D-26-14913

Title: Cryptic diversity in Astyanax (Characiformes: Acestrorhamphidae) from the Magdalena basin, Colombia: Insights from molecular and morphometric evidence

Dear Dr. Nogueira,

We sincerely appreciate the careful evaluation of our manuscript and the detailed and constructive comments provided by the Academic Editor and the reviewers. We have revised the manuscript accordingly, and we believe that these changes have substantially improved its clarity and scientific quality.

Below, we provide a point-by-point response to all comments. The reviewers’ and editor’s comments are reproduced in bold, followed by our responses. All changes are indicated in the revised manuscript with track changes.

We hope that the revised manuscript is now suitable for publication in PLOS ONE.

Sincerely,

Edna J. Márquez

---

Responses to the Journal Requirements

Journal Requirement 1: Please ensure that your manuscript meets PLOS ONE’s style requirements, including those for file naming.

Response: We have reviewed the manuscript and accompanying files to ensure compliance with PLOS ONE’s formatting and file-naming requirements.

Journal Requirement 2: Please review PLOS ONE’s guidelines on sharing author-generated code and ensure that the code supporting the findings is made available without restrictions.

Response: No original author-generated code was developed specifically for this study.

Journal Requirement 3: Please provide an amended Funding Statement declaring all funding or sources of support and include the statement: “There was no additional external funding received for this study.”

Response: We have amended the Funding Statement and included the complete updated wording in the cover letter, as requested. The revised Funding Statement reads:

“This study was supported by a grant framed under the project ‘Variabilidad genética de un banco de peces de los sectores medio y bajo del río Cauca’ (CT-2019-000661), funded by Empresas Públicas de Medellín and Universidad Nacional de Colombia, Sede Medellín. The funders had no role in the study design, data collection and analysis, decision to publish, or preparation of the manuscript. There was no additional external funding received for this study.”

Journal Requirement 4: Figure 1 appears to contain map or satellite imagery that may be copyrighted. Please provide permission or replace the figure with one that complies with the CC BY 4.0 license.

Response: We have replaced Figure 1 with a new map prepared in QGIS using nonproprietary and appropriately licensed or public-domain geographic data. The revised figure does not contain Google Maps, Google Earth, proprietary satellite imagery, or proprietary basemaps.

We also revised the figure legend to identify the geographic data sources as follows:

“Fig 1. Geographic location of the sampling sectors in the Cauca River basin, Colombia. Blue lines represent upstream and dam-proximate sectors (S1, PHI, S2, S3, and S4), in which genetic stock Asp1 predominated, while orange lines indicate downstream sectors (S5, S6, S7, and S8), in which genetic stock Asp2 predominated (see Results). Map layers: Colombia boundary from DANE Marco Geoestadístico Nacional (2024), available at https://geoportal.dane.gov.co/servicios/descarga-y-metadatos/datos-geoestadisticos/; inset boundaries from Natural Earth Admin 0 Countries (scale 1:10m), available at https://www.naturalearthdata.com/downloads/10m-cultural-vectors/10m-admin-0-countries/; rivers from HydroRIVERS v1.0/HydroSHEDS (15 arc-second resolution), available at https://www.hydrosheds.org/products/hydrorivers; elevation and hillshade from the HydroSHEDS void-filled DEM for South America (3 arc-second resolution), available at https://www.hydrosheds.org/hydrosheds-core-downloads; and physical background from Natural Earth II high-resolution raster data (scale 1:10m), available at https://www.naturalearthdata.com/downloads/10m-raster-data/10m-natural-earth-2/. Map created in QGIS without proprietary satellite imagery or basemaps.”

Journal Requirement 5: Please evaluate any references recommended by the reviewers and cite them only if they are relevant.

Response: We evaluated all publications recommended by the reviewers and incorporated those that were directly relevant to the interpretation and discussion of our results. The corresponding changes are described in our responses to Reviewer 1 below.

Journal Requirement 6: Please review the reference list to ensure that it is complete and correct and address any retracted publications.

Response: We carefully reviewed the reference list for completeness and accuracy. We also verified the status of the cited publications and found no retracted articles requiring removal or specific justification.

---

Response to the Academic Editor

The manuscript was evaluated by two expert reviewers, both of whom recognized the relevance of the research question, the robustness of the methodological approaches employed, and the overall quality of the results presented. Before a final decision can be made, the authors should address the comments and suggestions provided by the reviewers.

Response: We thank the Academic Editor for the positive assessment of our study. We have carefully considered and addressed all comments raised by the reviewers. The manuscript has been revised accordingly, and a detailed description of each modification is provided below.

---

Responses to Reviewer 1

We sincerely thank Reviewer 1 for the careful evaluation of our manuscript, the positive assessment of its scientific contribution, and the constructive suggestions.

Comment 1: In relation to the mitochondrial COI similarity between A. microlepis and Asp1, I suggest that the authors include this possibility in the Discussion. This would bring more robustness to the results. Barcoding analyses do not always provide conclusive results in morphologically cryptic species and complex groups such as Astyanax and Psalidodon. Low COI divergence may result from recent evolutionary radiation and other taxonomic and evolutionary processes.

Response: We agree with the reviewer that the mitochondrial similarity between A. microlepis and Asp1 deserves further consideration. We have expanded the Discussion to acknowledge that low COI divergence among closely related lineages may result from relatively recent evolutionary radiation and incomplete sorting of mitochondrial lineages.

The revised text reads:

“Consistent with this interpretation, low mitochondrial divergence based on COI barcodes has been reported among closely related lineages of Astyanax, a pattern that may reflect relatively recent evolutionary radiation and incomplete sorting of mitochondrial lineages [17].”

We evaluated the references suggested by the reviewer and incorporated those that were directly relevant to Astyanax and to the interpretation of our results.

Comment 2 — Line 61: "this is not the correct reference"

Response: The text has been corrected as suggested. The revised reference 8 in the list is:

“Toledo-Piza M, Baena EG, Dagosta FCP, Menezes NA, Ândrade M, Benine RC, et al. Checklist of the species of the Order Characiformes (Teleostei: Ostariophysi). Neotrop Ichthyol. 2024;22(1). doi:10.1590/1982-0224-2023-0086.”

Comment 3 — Line 71: "s in Astyanax´s, not in italic"

Response: The text has been corrected as suggested.

Comment 4: The name Psalidodon fasciatus should be used only for populations from the type locality in the São Francisco River basin.

Response: We thank the reviewer for this important clarification. We agree that the name Psalidodon fasciatus should be restricted to populations from its type locality in the São Francisco River basin.

Our original sentence was intended to describe the geographic distribution of species currently assigned to Psalidodon as a whole, rather than to suggest that P. fasciatus occurs throughout all the river basins mentioned. To remove this ambiguity, we revised the passage as follows:

“This species had traditionally been identified as Astyanax fasciatus [7] until this nominal species was reallocated to the recently resurrected genus Psalidodon. Species currently assigned to Psalidodon are geographically restricted to South American river basins, including the Rio São Francisco, the type locality of P. fasciatus, and the Paraná–Paraguay basin [2,5].”

---

Responses to Reviewer 2

We sincerely thank Reviewer 2 for the positive assessment of our manuscript and for the helpful suggestions.

Comment 1: Page 11, line 50: The genus does not occur as far south as Patagonia. The southernmost records of Astyanax, under its current circumscription, are A. abramis and A. lacustris in the Río de la Plata estuary in Argentina.

Response: We thank the reviewer for identifying this geographic inaccuracy. We have revised the sentence accordingly. It now reads:

“...extending from Texas to the Río de la Plata estuary in Argentina.”

Comment 2: Page 28, second paragraph, line 8: Change “configuration” to “shape.”

Response: We have replaced “configuration” with “shape,” as suggested.

Comment 3: Page 44, Figure 2: The quality of the figure is not good and needs to be improved.

Response: We have replaced Figure 2 with a higher-resolution version and improved the legibility of its labels and graphical elements.

Attachment Submitted filename: Response to reviewers.docx

# Peer review: Decision Letter 1

**DOI:** 10.1371/journal.pone.0347004.r003

**Authors:** Caio Nogueira

1 Sep 2026

Cryptic diversity in Astyanax (Characiformes: Acestrorhamphidae) from the Magdalena basin, Colombia: Insights from molecular and morphometric evidence

PONE-D-26-14913R1

Dear Dr. Márquez,

We’re pleased to inform you that your manuscript has been judged scientifically suitable for publication and will be formally accepted for publication once it meets all outstanding technical requirements.

Within one week, you’ll receive an e-mail detailing the required amendments. When these have been addressed, you’ll receive a formal acceptance letter and your manuscript will be scheduled for publication.

An invoice will be generated when your article is formally accepted. Please note, if your institution has a publishing partnership with PLOS and your article meets the relevant criteria, all or part of your publication costs will be covered. Please make sure your user information is up-to-date by logging into Editorial Manager at Editorial Manager® and clicking the ‘Update My Information' link at the top of the page. For questions related to billing, please contact billing support.

If your institution or institutions have a press office, please notify them about your upcoming paper to help maximize its impact. If they’ll be preparing press materials, please inform our press team as soon as possible -- no later than 48 hours after receiving the formal acceptance. Your manuscript will remain under strict press embargo until 2 pm Eastern Time on the date of publication. For more information, please contact onepress@plos.org.

Kind regards,

Caio Santos Nogueira, PhD

Academic Editor

PLOS One

Additional Editor Comments (optional):

Dear Authors,

One of the original reviewers has evaluated the revised version of the manuscript and indicated that the manuscript is now suitable for publication. Therefore, I would like to congratulate you on the work you have done and on the improvements made to the manuscript.

Reviewers' comments:

Reviewer's Responses to Questions

**Comments to the Author**

Reviewer #1: All comments have been addressed

**********

2. Is the manuscript technically sound, and do the data support the conclusions?

Reviewer #1: Yes

**********

3. Has the statistical analysis been performed appropriately and rigorously?

Reviewer #1: Yes

**********

4. Have the authors made all data underlying the findings in their manuscript fully available?

The PLOS Data policy

Reviewer #1: Yes

**********

5. Is the manuscript presented in an intelligible fashion and written in standard English?

Reviewer #1: Yes

**********

Reviewer #1: All suggestions made by the reviewers have been incorporated into the text. The manuscript is now more fluid and concise. Consequently, the manuscript is ready for publication.

**********

what does this mean?). If published, this will include your full peer review and any attached files.

If you choose “no”, your identity will remain anonymous but your review may still be made public.

**Do you want your identity to be public for this peer review?**  For information about this choice, including consent withdrawal, please see our Privacy Policy

Reviewer #1: No

**********

# Editor report: Acceptance letter

**DOI:** 10.1371/journal.pone.0347004.r004

**Authors:** Caio Nogueira

PONE-D-26-14913R1

PLOS One

Dear Dr. Márquez,

I'm pleased to inform you that your manuscript has been deemed suitable for publication in PLOS One. Congratulations! Your manuscript is now being handed over to our production team.

At this stage, our production department will prepare your paper for publication. This includes ensuring the following:

* All references, tables, and figures are properly cited

* All relevant supporting information is included in the manuscript submission,

* There are no issues that prevent the paper from being properly typeset

You will receive further instructions from the production team, including instructions on how to review your proof when it is ready. Please keep in mind that we are working through a large volume of accepted articles, so please give us a few days to review your paper and let you know the next and final steps.

Lastly, if your institution or institutions have a press office, please let them know about your upcoming paper now to help maximize its impact. If they'll be preparing press materials, please inform our press team within the next 48 hours. Your manuscript will remain under strict press embargo until 2 pm Eastern Time on the date of publication. For more information, please contact onepress@plos.org.

You will receive an invoice from PLOS for your publication fee after your manuscript has reached the completed accept phase. If you receive an email requesting payment before acceptance or for any other service, this may be a phishing scheme. Learn how to identify phishing emails and protect your accounts at https://explore.plos.org/phishing.

If we can help with anything else, please email us at customercare@plos.org.

Thank you for submitting your work to PLOS One and supporting open access.

Kind regards,

PLOS One Editorial Office Staff

on behalf of

Dr. Caio Santos Nogueira

Academic Editor

PLOS One
